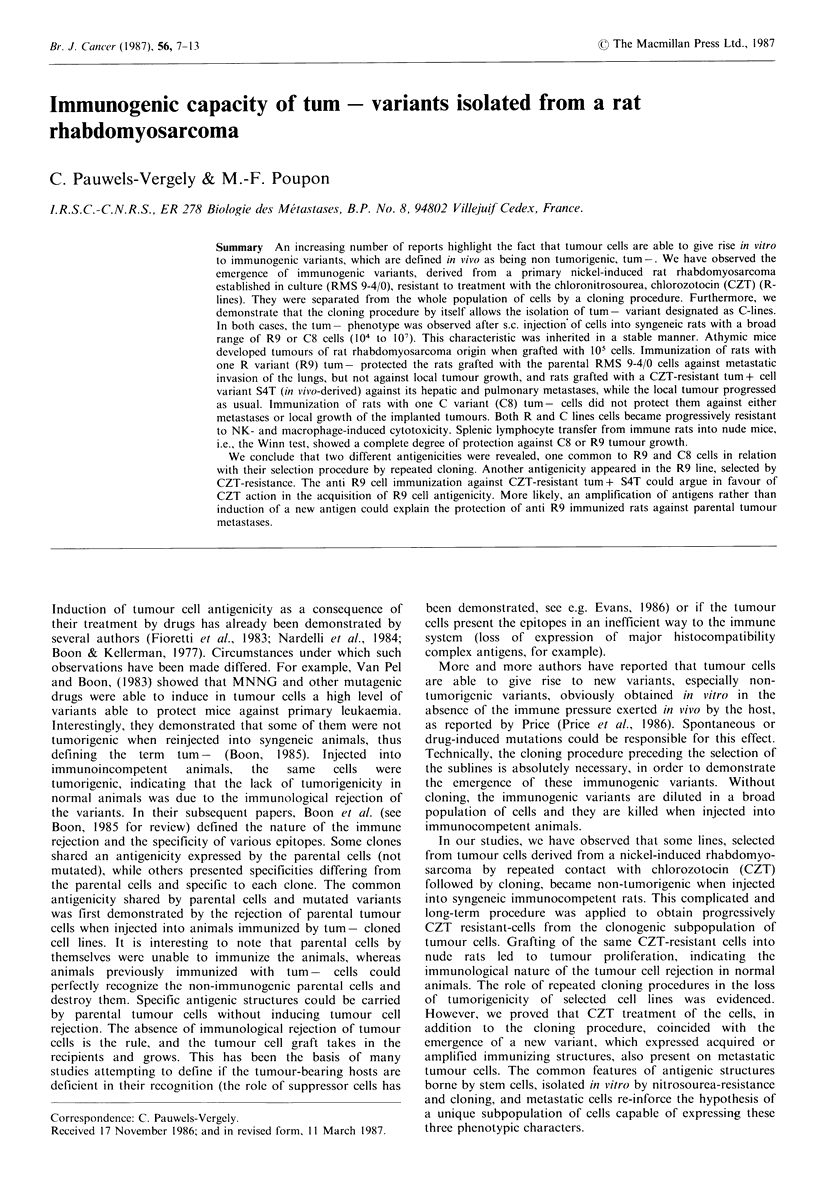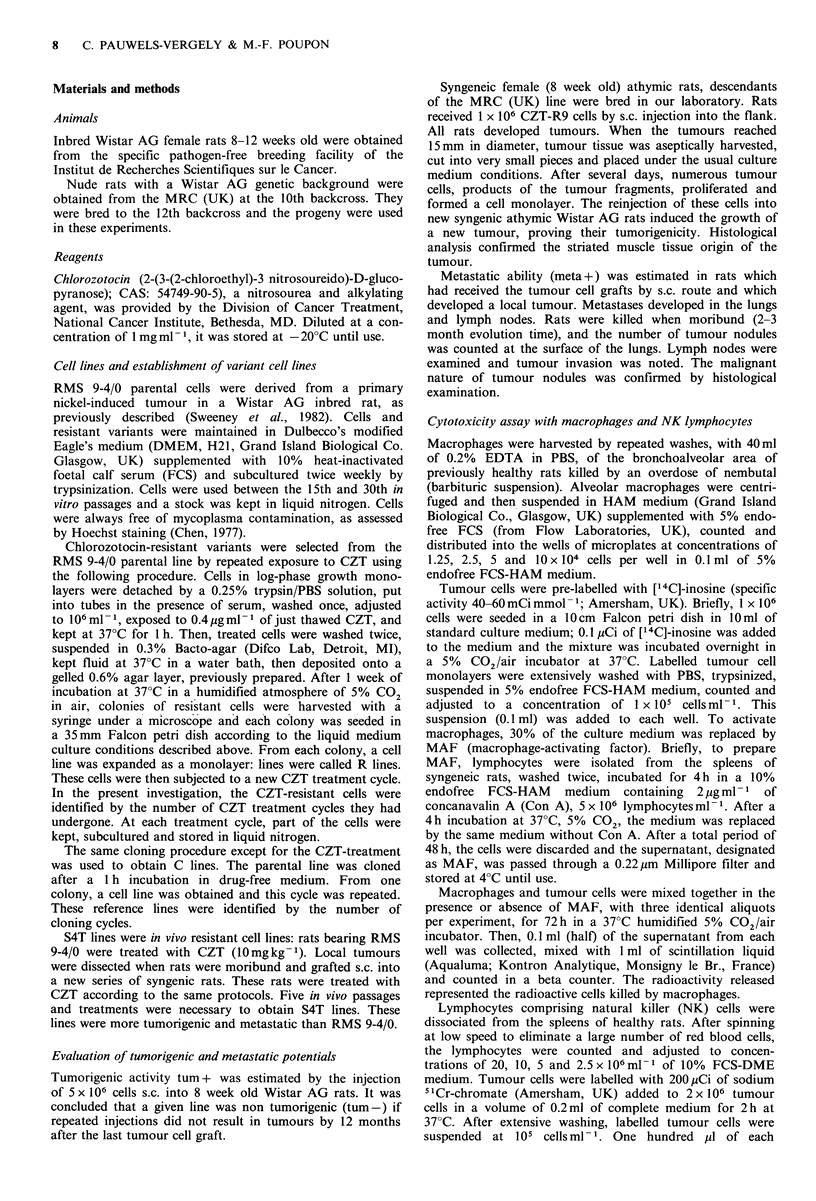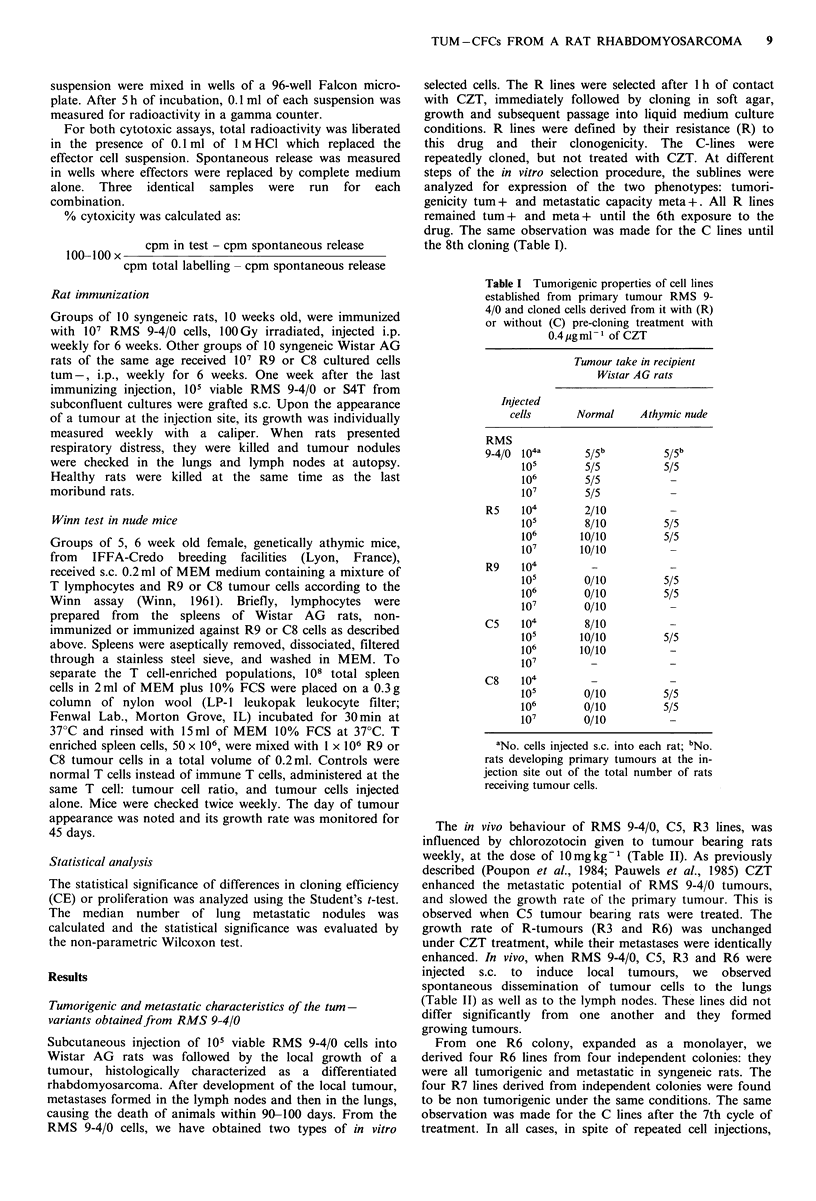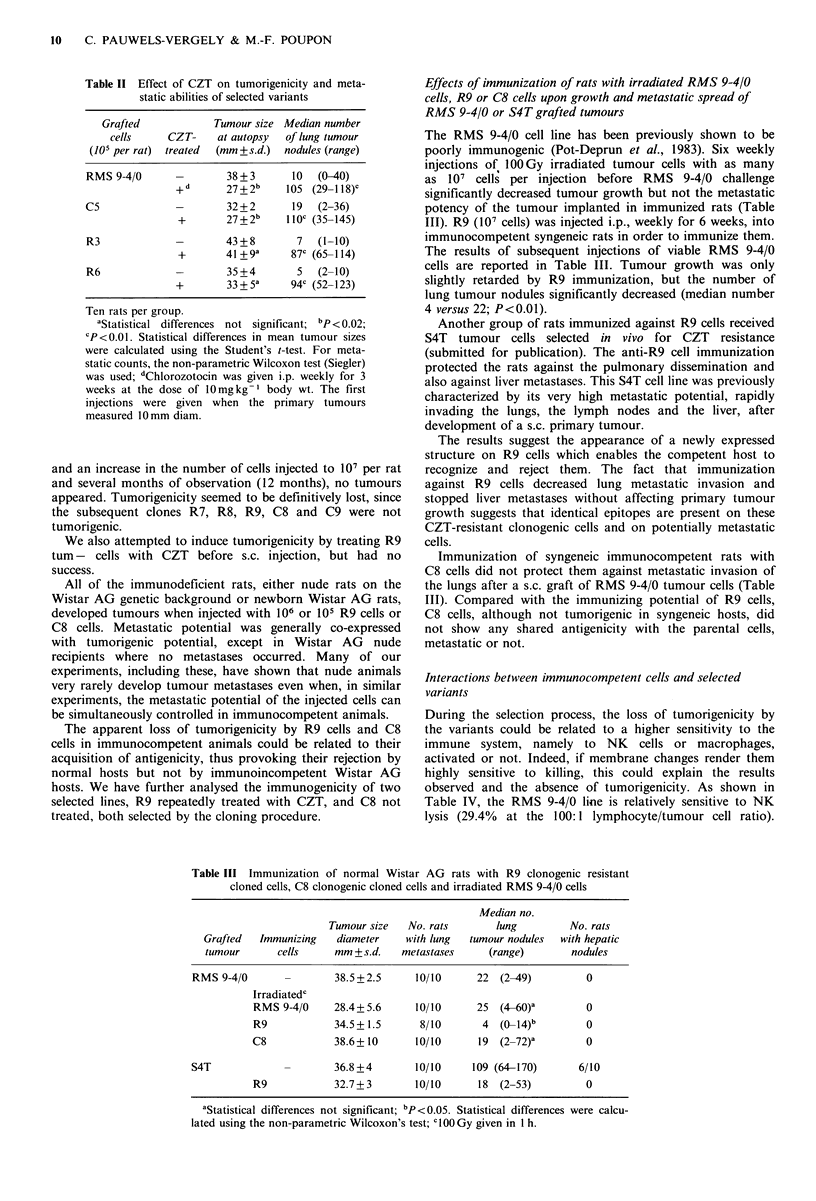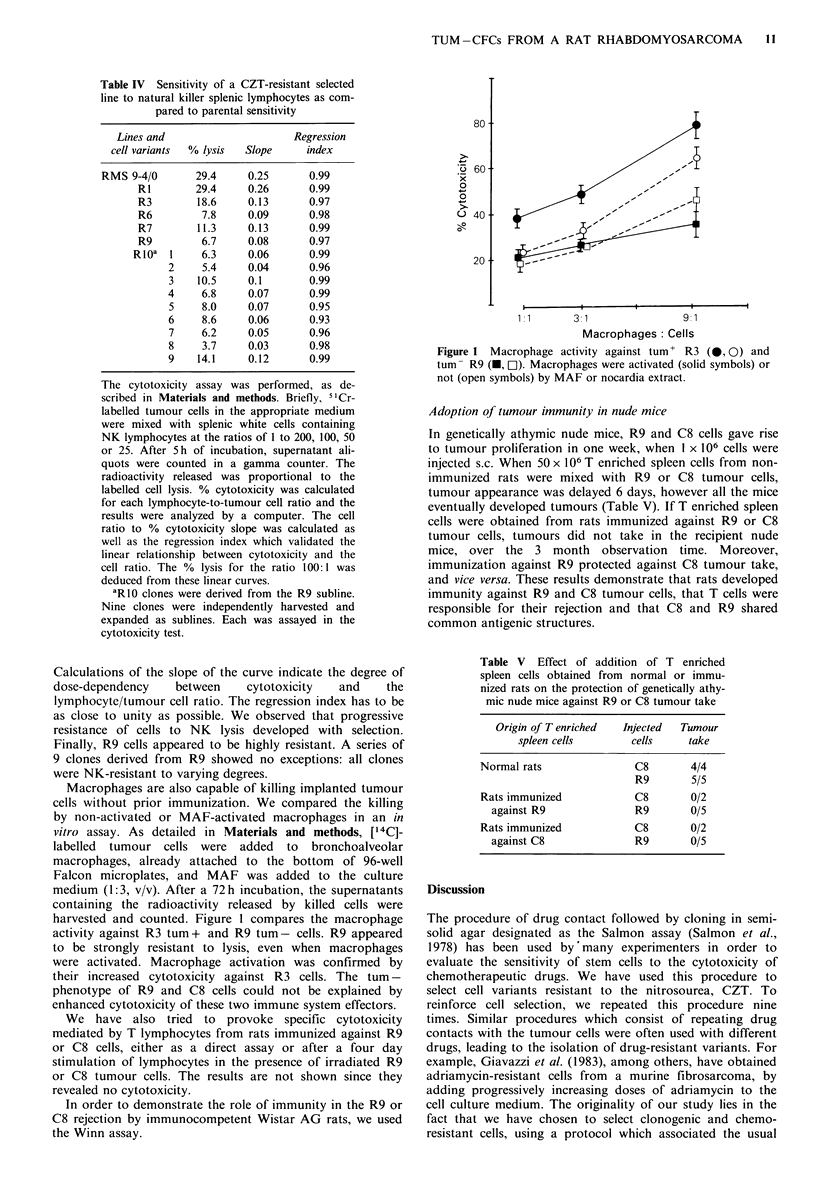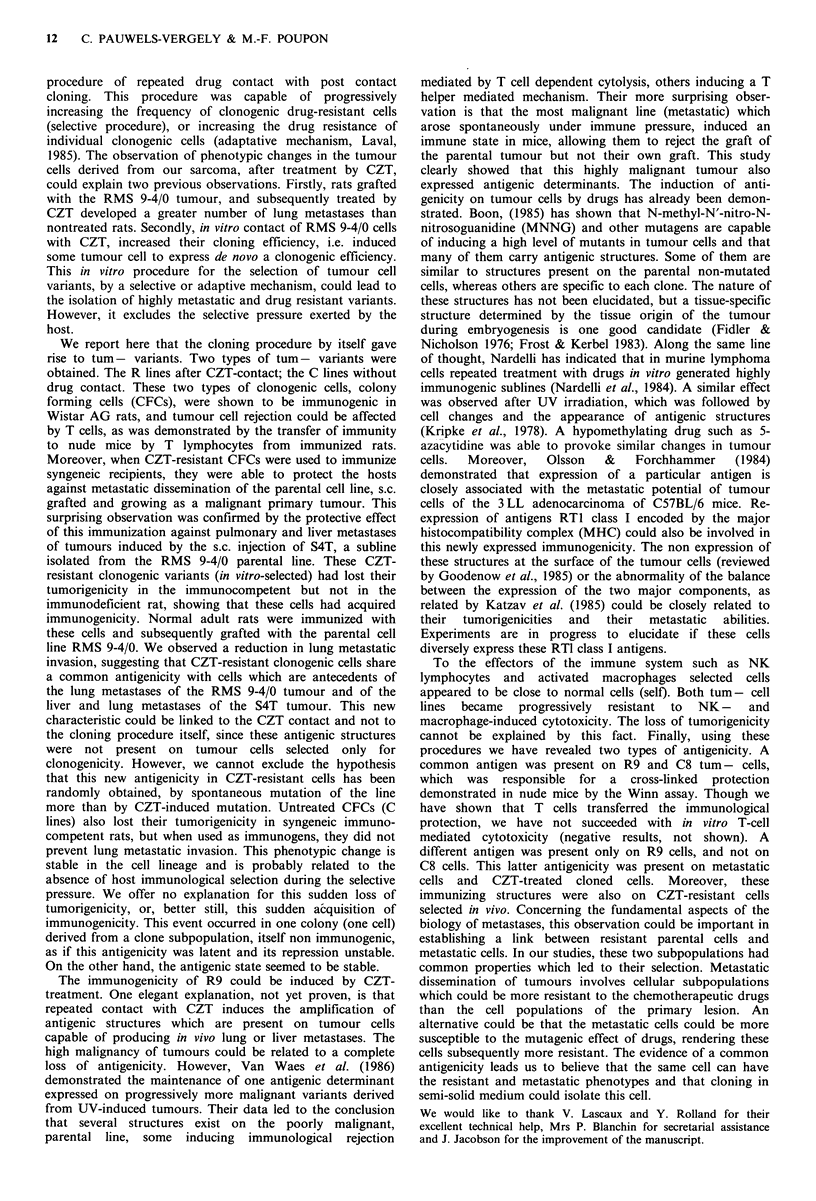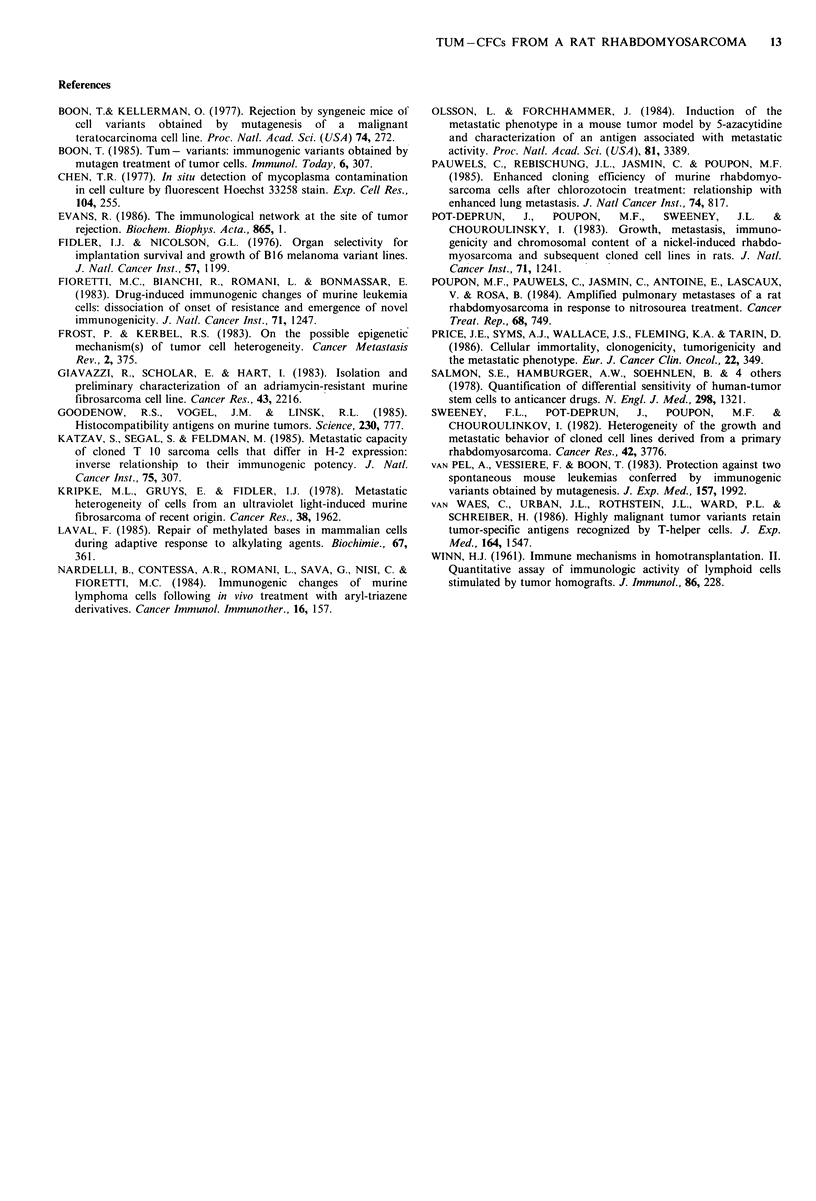# Immunogenic capacity of tum--variants isolated from a rat rhabdomyosarcoma.

**DOI:** 10.1038/bjc.1987.144

**Published:** 1987-07

**Authors:** C. Pauwels-Vergely, M. F. Poupon

## Abstract

An increasing number of reports highlight the fact that tumour cells are able to give rise in vitro to immunogenic variants, which are defined in vivo as being non tumorigenic, tum-. We have observed the emergence of immunogenic variants, derived from a primary nickel-induced rat rhabdomyosarcoma established in culture (RMS 9-4/0), resistant to treatment with the chloronitrosourea, chlorozotocin (CZT) (R-lines). They were separated from the whole population of cells by a cloning procedure. Furthermore, we demonstrate that the cloning procedure by itself allows the isolation of tum- variant designated as C-lines. In both cases, the tum- phenotype was observed after s.c. injection of cells into syngeneic rats with a broad range of R9 or C8 cells (10(4) to 10(7). This characteristic was inherited in a stable manner. Athymic mice developed tumours of rat rhabdomyosarcoma origin when grafted with 10(5) cells. Immunization of rats with one R variant (R9) tum- protected the rats grafted with the parental RMS 9-4/0 cells against metastatic invasion of the lungs, but not against local tumour growth, and rats grafted with a CZT-resistant tum+ cell variant S4T (in vivo-derived) against its hepatic and pulmonary metastases, while the local tumour progressed as usual. Immunization of rats with one C variant (C8) tum- cells did not protect them against either metastases or local growth of the implanted tumours. Both R and C lines cells became progressively resistant to NK- and macrophage-induced cytotoxicity. Splenic lymphocyte transfer from immune rats into nude mice, i.e., the Winn test, showed a complete degree of protection against C8 or R9 tumour growth. We conclude that two different antigenicities were revealed, one common to R9 and C8 cells in relation with their selection procedure by repeated cloning. Another antigenicity appeared in the R9 line, selected by CZT-resistance. The anti R9 cell immunization against CZT-resistant tum+ S4T could argue in favour of CZT action in the acquisition of R9 cell antigenicity. More likely, an amplification of antigens rather than induction of a new antigen could explain the protection of anti R9 immunized rats against parental tumour metastases.


Br. J. Cancer (1987), 56, 7–13 © The Macmillan Press Ltd., 1987

### Immunogenic capacity of tum – variants isolated from a rat rhabdomyosarcoma

C. Pauwels-Vergely & M.-F. Poupon

*I.R.S.C.-C.N.R.S., ER 278 Biologie des Métastases, B.P. No. 8, 94802 Villejuif Cedex, France.*

**Summary** An increasing number of reports highlight the fact that tumour cells are able to give rise *in vitro* to immunogenic variants, which are defined *in vivo* as being non tumorigenic, tum –. We have observed the emergence of immunogenic variants, derived from a primary nickel-induced rat rhabdomyosarcoma established in culture (RMS 9-4/0), resistant to treatment with the chloronitrosourea, chlorozotocin (CZT) (R-lines). They were separated from the whole population of cells by a cloning procedure. Furthermore, we demonstrate that the cloning procedure by itself allows the isolation of tum – variant designated as C-lines. In both cases, the tum – phenotype was observed after s.c. injection of cells into syngeneic rats with a broad range of R9 or C8 cells ($10^4$ to $10^7$). This characteristic was inherited in a stable manner. Athymic mice developed tumours of rat rhabdomyosarcoma origin when grafted with $10^5$ cells. Immunization of rats with one R variant (R9) tum – protected the rats grafted with the parental RMS 9-4/0 cells against metastatic invasion of the lungs, but not against local tumour growth, and rats grafted with a CZT-resistant tum + cell variant S4T (*in vivo*-derived) against its hepatic and pulmonary metastases, while the local tumour progressed as usual. Immunization of rats with one C variant (C8) tum – cells did not protect them against either metastases or local growth of the implanted tumours. Both R and C lines cells became progressively resistant to NK- and macrophage-induced cytotoxicity. Splenic lymphocyte transfer from immune rats into nude mice, i.e., the Winn test, showed a complete degree of protection against C8 or R9 tumour growth.

We conclude that two different antigenicities were revealed, one common to R9 and C8 cells in relation with their selection procedure by repeated cloning. Another antigenicity appeared in the R9 line, selected by CZT-resistance. The anti R9 cell immunization against CZT-resistant tum + S4T could argue in favour of CZT action in the acquisition of R9 cell antigenicity. More likely, an amplification of antigens rather than induction of a new antigen could explain the protection of anti R9 immunized rats against parental tumour metastases.

Induction of tumour cell antigenicity as a consequence of their treatment by drugs has already been demonstrated by several authors (Fioretti *et al.*, 1983; Nardelli *et al.*, 1984; Boon & Kellerman, 1977). Circumstances under which such observations have been made differed. For example, Van Pel and Boon, (1983) showed that MNNG and other mutagenic drugs were able to induce in tumour cells a high level of variants able to protect mice against primary leukaemia. Interestingly, they demonstrated that some of them were not tumorigenic when reinjected into syngeneic animals, thus defining the term tum – (Boon, 1985). Injected into immunoincompetent animals, the same cells were tumorigenic, indicating that the lack of tumorigenicity in normal animals was due to the immunological rejection of the variants. In their subsequent papers, Boon *et al.* (see Boon, 1985 for review) defined the nature of the immune rejection and the specificity of various epitopes. Some clones shared an antigenicity expressed by the parental cells (not mutated), while others presented specificities differing from the parental cells and specific to each clone. The common antigenicity shared by parental cells and mutated variants was first demonstrated by the rejection of parental tumour cells when injected into animals immunized by tum – cloned cell lines. It is interesting to note that parental cells by themselves were unable to immunize the animals, whereas animals previously immunized with tum – cells could perfectly recognize the non-immunogenic parental cells and destroy them. Specific antigenic structures could be carried by parental tumour cells without inducing tumour cell rejection. The absence of immunological rejection of tumour cells is the rule, and the tumour cell graft takes in the recipients and grows. This has been the basis of many studies attempting to define if the tumour-bearing hosts are deficient in their recognition (the role of suppressor cells has been demonstrated, see e.g. Evans, 1986) or if the tumour cells present the epitopes in an inefficient way to the immune system (loss of expression of major histocompatibility complex antigens, for example).

More and more authors have reported that tumour cells are able to give rise to new variants, especially non-tumorigenic variants, obviously obtained *in vitro* in the absence of the immune pressure exerted *in vivo* by the host, as reported by Price (Price *et al.*, 1986). Spontaneous or drug-induced mutations could be responsible for this effect. Technically, the cloning procedure preceding the selection of the sublines is absolutely necessary, in order to demonstrate the emergence of these immunogenic variants. Without cloning, the immunogenic variants are diluted in a broad population of cells and they are killed when injected into immunocompetent animals.

In our studies, we have observed that some lines, selected from tumour cells derived from a nickel-induced rhabdomyosarcoma by repeated contact with chlorozotocin (CZT) followed by cloning, became non-tumorigenic when injected into syngeneic immunocompetent rats. This complicated and long-term procedure was applied to obtain progressively CZT resistant-cells from the clonogenic subpopulation of tumour cells. Grafting of the same CZT-resistant cells into nude rats led to tumour proliferation, indicating the immunological nature of the tumour cell rejection in normal animals. The role of repeated cloning procedures in the loss of tumorigenicity of selected cell lines was evidenced. However, we proved that CZT treatment of the cells, in addition to the cloning procedure, coincided with the emergence of a new variant, which expressed acquired or amplified immunizing structures, also present on metastatic tumour cells. The common features of antigenic structures borne by stem cells, isolated *in vitro* by nitrosourea-resistance and cloning, and metastatic cells re-inforce the hypothesis of a unique subpopulation of cells capable of expressing these three phenotypic characters.

Correspondence: C. Pauwels-Vergely.
Received 17 November 1986; and in revised form, 11 March 1987.

### Materials and methods

### *Animals*

Inbred Wistar AG female rats 8–12 weeks old were obtained from the specific pathogen-free breeding facility of the Institut de Recherches Scientifiques sur le Cancer.

Nude rats with a Wistar AG genetic background were obtained from the MRC (UK) at the 10th backcross. They were bred to the 12th backcross and the progeny were used in these experiments.

### *Reagents*

*Chlorozotocin* (2-(3-(2-chloroethyl)-3 nitrosoureido)-D-glucopyranose); CAS: 54749-90-5), a nitrosourea and alkylating agent, was provided by the Division of Cancer Treatment, National Cancer Institute, Bethesda, MD. Diluted at a concentration of $1\,mg\,ml^{-1}$, it was stored at $-20°C$ until use.

### *Cell lines and establishment of variant cell lines*

RMS 9-4/0 parental cells were derived from a primary nickel-induced tumour in a Wistar AG inbred rat, as previously described (Sweeney *et al.*, 1982). Cells and resistant variants were maintained in Dulbecco's modified Eagle's medium (DMEM, H21, Grand Island Biological Co. Glasgow, UK) supplemented with 10% heat-inactivated foetal calf serum (FCS) and subcultured twice weekly by trypsinization. Cells were used between the 15th and 30th *in vitro* passages and a stock was kept in liquid nitrogen. Cells were always free of mycoplasma contamination, as assessed by Hoechst staining (Chen, 1977).

Chlorozotocin-resistant variants were selected from the RMS 9-4/0 parental line by repeated exposure to CZT using the following procedure. Cells in log-phase growth monolayers were detached by a 0.25% trypsin/PBS solution, put into tubes in the presence of serum, washed once, adjusted to $10^6\,ml^{-1}$, exposed to $0.4\,\mu g\,ml^{-1}$ of just thawed CZT, and kept at 37°C for 1 h. Then, treated cells were washed twice, suspended in 0.3% Bacto-agar (Difco Lab, Detroit, MI), kept fluid at 37°C in a water bath, then deposited onto a gelled 0.6% agar layer, previously prepared. After 1 week of incubation at 37°C in a humidified atmosphere of 5% $CO_2$ in air, colonies of resistant cells were harvested with a syringe under a microscope and each colony was seeded in a 35 mm Falcon petri dish according to the liquid medium culture conditions described above. From each colony, a cell line was expanded as a monolayer: lines were called R lines. These cells were then subjected to a new CZT treatment cycle. In the present investigation, the CZT-resistant cells were identified by the number of CZT treatment cycles they had undergone. At each treatment cycle, part of the cells were kept, subcultured and stored in liquid nitrogen.

The same cloning procedure except for the CZT-treatment was used to obtain C lines. The parental line was cloned after a 1 h incubation in drug-free medium. From one colony, a cell line was obtained and this cycle was repeated. These reference lines were identified by the number of cloning cycles.

S4T lines were *in vivo* resistant cell lines: rats bearing RMS 9-4/0 were treated with CZT ($10\,mg\,kg^{-1}$). Local tumours were dissected when rats were moribund and grafted s.c. into a new series of syngenic rats. These rats were treated with CZT according to the same protocols. Five *in vivo* passages and treatments were necessary to obtain S4T lines. These lines were more tumorigenic and metastatic than RMS 9-4/0.

### *Evaluation of tumorigenic and metastatic potentials*

Tumorigenic activity tum+ was estimated by the injection of $5\times10^6$ cells s.c. into 8 week old Wistar AG rats. It was concluded that a given line was non tumorigenic (tum−) if repeated injections did not result in tumours by 12 months after the last tumour cell graft.

Syngeneic female (8 week old) athymic rats, descendants of the MRC (UK) line were bred in our laboratory. Rats received $1\times10^6$ CZT-R9 cells by s.c. injection into the flank. All rats developed tumours. When the tumours reached 15 mm in diameter, tumour tissue was aseptically harvested, cut into very small pieces and placed under the usual culture medium conditions. After several days, numerous tumour cells, products of the tumour fragments, proliferated and formed a cell monolayer. The reinjection of these cells into new syngenic athymic Wistar AG rats induced the growth of a new tumour, proving their tumorigenicity. Histological analysis confirmed the striated muscle tissue origin of the tumour.

Metastatic ability (meta+) was estimated in rats which had received the tumour cell grafts by s.c. route and which developed a local tumour. Metastases developed in the lungs and lymph nodes. Rats were killed when moribund (2–3 month evolution time), and the number of tumour nodules was counted at the surface of the lungs. Lymph nodes were examined and tumour invasion was noted. The malignant nature of tumour nodules was confirmed by histological examination.

### *Cytotoxicity assay with macrophages and NK lymphocytes*

Macrophages were harvested by repeated washes, with 40 ml of 0.2% EDTA in PBS, of the bronchoalveolar area of previously healthy rats killed by an overdose of nembutal (barbituric suspension). Alveolar macrophages were centrifuged and then suspended in HAM medium (Grand Island Biological Co., Glasgow, UK) supplemented with 5% endofree FCS (from Flow Laboratories, UK), counted and distributed into the wells of microplates at concentrations of 1.25, 2.5, 5 and $10\times10^4$ cells per well in 0.1 ml of 5% endofree FCS-HAM medium.

Tumour cells were pre-labelled with [$^{14}$C]-inosine (specific activity 40–60 $mCi\,mmol^{-1}$; Amersham, UK). Briefly, $1\times10^6$ cells were seeded in a 10 cm Falcon petri dish in 10 ml of standard culture medium; 0.1 $\mu$Ci of [$^{14}$C]-inosine was added to the medium and the mixture was incubated overnight in a 5% $CO_2$/air incubator at 37°C. Labelled tumour cell monolayers were extensively washed with PBS, trypsinized, suspended in 5% endofree FCS-HAM medium, counted and adjusted to a concentration of $1\times10^5$ $cells\,ml^{-1}$. This suspension (0.1 ml) was added to each well. To activate macrophages, 30% of the culture medium was replaced by MAF (macrophage-activating factor). Briefly, to prepare MAF, lymphocytes were isolated from the spleens of syngeneic rats, washed twice, incubated for 4 h in a 10% endofree FCS-HAM medium containing $2\,\mu g\,ml^{-1}$ of concanavalin A (Con A), $5\times10^6$ $lymphocytes\,ml^{-1}$. After a 4 h incubation at 37°C, 5% $CO_2$, the medium was replaced by the same medium without Con A. After a total period of 48 h, the cells were discarded and the supernatant, designated as MAF, was passed through a 0.22 $\mu$m Millipore filter and stored at 4°C until use.

Macrophages and tumour cells were mixed together in the presence or absence of MAF, with three identical aliquots per experiment, for 72 h in a 37°C humidified 5% $CO_2$/air incubator. Then, 0.1 ml (half) of the supernatant from each well was collected, mixed with 1 ml of scintillation liquid (Aqualuma; Kontron Analytique, Monsigny le Br., France) and counted in a beta counter. The radioactivity released represented the radioactive cells killed by macrophages.

Lymphocytes comprising natural killer (NK) cells were dissociated from the spleens of healthy rats. After spinning at low speed to eliminate a large number of red blood cells, the lymphocytes were counted and adjusted to concentrations of 20, 10, 5 and $2.5\times10^6\,ml^{-1}$ of 10% FCS-DME medium. Tumour cells were labelled with 200 $\mu$Ci of sodium $^{51}$Cr-chromate (Amersham, UK) added to $2\times10^6$ tumour cells in a volume of 0.2 ml of complete medium for 2 h at 37°C. After extensive washing, labelled tumour cells were suspended at $10^5$ $cells\,ml^{-1}$. One hundred $\mu$l of each suspension were mixed in wells of a 96-well Falcon microplate. After 5 h of incubation, 0.1 ml of each suspension was measured for radioactivity in a gamma counter.

For both cytotoxic assays, total radioactivity was liberated in the presence of 0.1 ml of 1 M HCl which replaced the effector cell suspension. Spontaneous release was measured in wells where effectors were replaced by complete medium alone. Three identical samples were run for each combination.

% cytoxicity was calculated as:

$$100\text{–}100 \times \frac{\text{cpm in test} - \text{cpm spontaneous release}}{\text{cpm total labelling} - \text{cpm spontaneous release}}$$

*Rat immunization*

Groups of 10 syngeneic rats, 10 weeks old, were immunized with $10^7$ RMS 9-4/0 cells, 100 Gy irradiated, injected i.p. weekly for 6 weeks. Other groups of 10 syngeneic Wistar AG rats of the same age received $10^7$ R9 or C8 cultured cells tum–, i.p., weekly for 6 weeks. One week after the last immunizing injection, $10^5$ viable RMS 9-4/0 or S4T from subconfluent cultures were grafted s.c. Upon the appearance of a tumour at the injection site, its growth was individually measured weekly with a caliper. When rats presented respiratory distress, they were killed and tumour nodules were checked in the lungs and lymph nodes at autopsy. Healthy rats were killed at the same time as the last moribund rats.

*Winn test in nude mice*

Groups of 5, 6 week old female, genetically athymic mice, from IFFA-Credo breeding facilities (Lyon, France), received s.c. 0.2 ml of MEM medium containing a mixture of T lymphocytes and R9 or C8 tumour cells according to the Winn assay (Winn, 1961). Briefly, lymphocytes were prepared from the spleens of Wistar AG rats, non-immunized or immunized against R9 or C8 cells as described above. Spleens were aseptically removed, dissociated, filtered through a stainless steel sieve, and washed in MEM. To separate the T cell-enriched populations, $10^8$ total spleen cells in 2 ml of MEM plus 10% FCS were placed on a 0.3 g column of nylon wool (LP-1 leukopak leukocyte filter; Fenwal Lab., Morton Grove, IL) incubated for 30 min at 37°C and rinsed with 15 ml of MEM 10% FCS at 37°C. T enriched spleen cells, $50 \times 10^6$, were mixed with $1 \times 10^6$ R9 or C8 tumour cells in a total volume of 0.2 ml. Controls were normal T cells instead of immune T cells, administered at the same T cell: tumour cell ratio, and tumour cells injected alone. Mice were checked twice weekly. The day of tumour appearance was noted and its growth rate was monitored for 45 days.

*Statistical analysis*

The statistical significance of differences in cloning efficiency (CE) or proliferation was analyzed using the Student's $t$-test. The median number of lung metastatic nodules was calculated and the statistical significance was evaluated by the non-parametric Wilcoxon test.

### Results

*Tumorigenic and metastatic characteristics of the tum– variants obtained from RMS 9-4/0*

Subcutaneous injection of $10^5$ viable RMS 9-4/0 cells into Wistar AG rats was followed by the local growth of a tumour, histologically characterized as a differentiated rhabdomyosarcoma. After development of the local tumour, metastases formed in the lymph nodes and then in the lungs, causing the death of animals within 90–100 days. From the RMS 9-4/0 cells, we have obtained two types of *in vitro* selected cells. The R lines were selected after 1 h of contact with CZT, immediately followed by cloning in soft agar, growth and subsequent passage into liquid medium culture conditions. R lines were defined by their resistance (R) to this drug and their clonogenicity. The C-lines were repeatedly cloned, but not treated with CZT. At different steps of the *in vitro* selection procedure, the sublines were analyzed for expression of the two phenotypes: tumorigenicity tum+ and metastatic capacity meta+. All R lines remained tum+ and meta+ until the 6th exposure to the drug. The same observation was made for the C lines until the 8th cloning (Table I).

**Table I** Tumorigenic properties of cell lines established from primary tumour RMS 9-4/0 and cloned cells derived from it with (R) or without (C) pre-cloning treatment with 0.4 $\mu$g ml$^{-1}$ of CZT

| Injected cells | | *Tumour take in recipient Wistar AG rats* Normal | *Athymic nude* |
|---|---|---|---|
| RMS 9-4/0 | $10^{4a}$ | 5/5[b] | 5/5[b] |
| | $10^5$ | 5/5 | 5/5 |
| | $10^6$ | 5/5 | – |
| | $10^7$ | 5/5 | – |
| R5 | $10^4$ | 2/10 | – |
| | $10^5$ | 8/10 | 5/5 |
| | $10^6$ | 10/10 | 5/5 |
| | $10^7$ | 10/10 | – |
| R9 | $10^4$ | – | – |
| | $10^5$ | 0/10 | 5/5 |
| | $10^6$ | 0/10 | 5/5 |
| | $10^7$ | 0/10 | – |
| C5 | $10^4$ | 8/10 | – |
| | $10^5$ | 10/10 | 5/5 |
| | $10^6$ | 10/10 | – |
| | $10^7$ | – | – |
| C8 | $10^4$ | – | – |
| | $10^5$ | 0/10 | 5/5 |
| | $10^6$ | 0/10 | 5/5 |
| | $10^7$ | 0/10 | – |

[a]No. cells injected s.c. into each rat; [b]No. rats developing primary tumours at the injection site out of the total number of rats receiving tumour cells.

The *in vivo* behaviour of RMS 9-4/0, C5, R3 lines, was influenced by chlorozotocin given to tumour bearing rats weekly, at the dose of 10 mg kg$^{-1}$ (Table II). As previously described (Poupon *et al.*, 1984; Pauwels *et al.*, 1985) CZT enhanced the metastatic potential of RMS 9-4/0 tumours, and slowed the growth rate of the primary tumour. This is observed when C5 tumour bearing rats were treated. The growth rate of R-tumours (R3 and R6) was unchanged under CZT treatment, while their metastases were identically enhanced. *In vivo*, when RMS 9-4/0, C5, R3 and R6 were injected s.c. to induce local tumours, we observed spontaneous dissemination of tumour cells to the lungs (Table II) as well as to the lymph nodes. These lines did not differ significantly from one another and they formed growing tumours.

From one R6 colony, expanded as a monolayer, we derived four R6 lines from four independent colonies: they were all tumorigenic and metastatic in syngeneic rats. The four R7 lines derived from independent colonies were found to be non tumorigenic under the same conditions. The same observation was made for the C lines after the 7th cycle of treatment. In all cases, in spite of repeated cell injections,

**Table II** Effect of CZT on tumorigenicity and metastatic abilities of selected variants

| *Grafted cells ($10^5$ per rat)* | *CZT-treated* | *Tumour size at autopsy (mm ± s.d.)* | *Median number of lung tumour nodules (range)* |
|---|---|---|---|
| RMS 9-4/0 | – | 38±3 | 10 (0–40) |
| | +[d] | 27±2[b] | 105 (29–118)[c] |
| C5 | – | 32±2 | 19 (2–36) |
| | + | 27±2[b] | 110[c] (35–145) |
| R3 | – | 43±8 | 7 (1–10) |
| | + | 41±9[a] | 87[c] (65–114) |
| R6 | – | 35±4 | 5 (2–10) |
| | + | 33±5[a] | 94[c] (52–123) |

Ten rats per group.
[a]Statistical differences not significant; [b]$P<0.02$; [c]$P<0.01$. Statistical differences in mean tumour sizes were calculated using the Student's *t*-test. For metastatic counts, the non-parametric Wilcoxon test (Siegler) was used; [d]Chlorozotocin was given i.p. weekly for 3 weeks at the dose of $10\,mg\,kg^{-1}$ body wt. The first injections were given when the primary tumours measured 10 mm diam.

and an increase in the number of cells injected to $10^7$ per rat and several months of observation (12 months), no tumours appeared. Tumorigenicity seemed to be definitively lost, since the subsequent clones R7, R8, R9, C8 and C9 were not tumorigenic.

We also attempted to induce tumorigenicity by treating R9 tum– cells with CZT before s.c. injection, but had no success.

All of the immunodeficient rats, either nude rats on the Wistar AG genetic background or newborn Wistar AG rats, developed tumours when injected with $10^6$ or $10^5$ R9 cells or C8 cells. Metastatic potential was generally co-expressed with tumorigenic potential, except in Wistar AG nude recipients where no metastases occurred. Many of our experiments, including these, have shown that nude animals very rarely develop tumour metastases even when, in similar experiments, the metastatic potential of the injected cells can be simultaneously controlled in immunocompetent animals.

The apparent loss of tumorigenicity by R9 cells and C8 cells in immunocompetent animals could be related to their acquisition of antigenicity, thus provoking their rejection by normal hosts but not by immunoincompetent Wistar AG hosts. We have further analysed the immunogenicity of two selected lines, R9 repeatedly treated with CZT, and C8 not treated, both selected by the cloning procedure.

### *Effects of immunization of rats with irradiated RMS 9-4/0 cells, R9 or C8 cells upon growth and metastatic spread of RMS 9-4/0 or S4T grafted tumours*

The RMS 9-4/0 cell line has been previously shown to be poorly immunogenic (Pot-Deprun *et al.*, 1983). Six weekly injections of 100 Gy irradiated tumour cells with as many as $10^7$ cells per injection before RMS 9-4/0 challenge significantly decreased tumour growth but not the metastatic potency of the tumour implanted in immunized rats (Table III). R9 ($10^7$ cells) was injected i.p., weekly for 6 weeks, into immunocompetent syngeneic rats in order to immunize them. The results of subsequent injections of viable RMS 9-4/0 cells are reported in Table III. Tumour growth was only slightly retarded by R9 immunization, but the number of lung tumour nodules significantly decreased (median number 4 *versus* 22; $P<0.01$).

Another group of rats immunized against R9 cells received S4T tumour cells selected *in vivo* for CZT resistance (submitted for publication). The anti-R9 cell immunization protected the rats against the pulmonary dissemination and also against liver metastases. This S4T cell line was previously characterized by its very high metastatic potential, rapidly invading the lungs, the lymph nodes and the liver, after development of a s.c. primary tumour.

The results suggest the appearance of a newly expressed structure on R9 cells which enables the competent host to recognize and reject them. The fact that immunization against R9 cells decreased lung metastatic invasion and stopped liver metastases without affecting primary tumour growth suggests that identical epitopes are present on these CZT-resistant clonogenic cells and on potentially metastatic cells.

Immunization of syngeneic immunocompetent rats with C8 cells did not protect them against metastatic invasion of the lungs after a s.c. graft of RMS 9-4/0 tumour cells (Table III). Compared with the immunizing potential of R9 cells, C8 cells, although not tumorigenic in syngeneic hosts, did not show any shared antigenicity with the parental cells, metastatic or not.

### *Interactions between immunocompetent cells and selected variants*

During the selection process, the loss of tumorigenicity by the variants could be related to a higher sensitivity to the immune system, namely to NK cells or macrophages, activated or not. Indeed, if membrane changes render them highly sensitive to killing, this could explain the results observed and the absence of tumorigenicity. As shown in Table IV, the RMS 9-4/0 line is relatively sensitive to NK lysis (29.4% at the 100:1 lymphocyte/tumour cell ratio).

**Table III** Immunization of normal Wistar AG rats with R9 clonogenic resistant cloned cells, C8 clonogenic cloned cells and irradiated RMS 9-4/0 cells

| *Grafted tumour* | *Immunizing cells* | *Tumour size diameter mm ± s.d.* | *No. rats with lung metastases* | *Median no. lung tumour nodules (range)* | *No. rats with hepatic nodules* |
|---|---|---|---|---|---|
| RMS 9-4/0 | – | 38.5±2.5 | 10/10 | 22 (2–49) | 0 |
| | Irradiated[c] RMS 9-4/0 | 28.4±5.6 | 10/10 | 25 (4–60)[a] | 0 |
| | R9 | 34.5±1.5 | 8/10 | 4 (0–14)[b] | 0 |
| | C8 | 38.6±10 | 10/10 | 19 (2–72)[a] | 0 |
| S4T | – | 36.8±4 | 10/10 | 109 (64–170) | 6/10 |
| | R9 | 32.7±3 | 10/10 | 18 (2–53) | 0 |

[a]Statistical differences not significant; [b]$P<0.05$. Statistical differences were calculated using the non-parametric Wilcoxon's test; [c]100 Gy given in 1 h.

**Table IV** Sensitivity of a CZT-resistant selected line to natural killer splenic lymphocytes as compared to parental sensitivity

| *Lines and cell variants* | | *% lysis* | *Slope* | *Regression index* |
|---|---|---|---|---|
| RMS 9-4/0 | | 29.4 | 0.25 | 0.99 |
| R1 | | 29.4 | 0.26 | 0.99 |
| R3 | | 18.6 | 0.13 | 0.97 |
| R6 | | 7.8 | 0.09 | 0.98 |
| R7 | | 11.3 | 0.13 | 0.99 |
| R9 | | 6.7 | 0.08 | 0.97 |
| R10[a] | 1 | 6.3 | 0.06 | 0.99 |
| | 2 | 5.4 | 0.04 | 0.96 |
| | 3 | 10.5 | 0.1 | 0.99 |
| | 4 | 6.8 | 0.07 | 0.99 |
| | 5 | 8.0 | 0.07 | 0.95 |
| | 6 | 8.6 | 0.06 | 0.93 |
| | 7 | 6.2 | 0.05 | 0.96 |
| | 8 | 3.7 | 0.03 | 0.98 |
| | 9 | 14.1 | 0.12 | 0.99 |

The cytotoxicity assay was performed, as described in **Materials and methods**. Briefly, $^{51}$Cr-labelled tumour cells in the appropriate medium were mixed with splenic white cells containing NK lymphocytes at the ratios of 1 to 200, 100, 50 or 25. After 5 h of incubation, supernatant aliquots were counted in a gamma counter. The radioactivity released was proportional to the labelled cell lysis. % cytotoxicity was calculated for each lymphocyte-to-tumour cell ratio and the results were analyzed by a computer. The cell ratio to % cytotoxicity slope was calculated as well as the regression index which validated the linear relationship between cytotoxicity and the cell ratio. The % lysis for the ratio 100:1 was deduced from these linear curves.

[a]R10 clones were derived from the R9 subline. Nine clones were independently harvested and expanded as sublines. Each was assayed in the cytotoxicity test.

Calculations of the slope of the curve indicate the degree of dose-dependency between cytotoxicity and the lymphocyte/tumour cell ratio. The regression index has to be as close to unity as possible. We observed that progressive resistance of cells to NK lysis developed with selection. Finally, R9 cells appeared to be highly resistant. A series of 9 clones derived from R9 showed no exceptions: all clones were NK-resistant to varying degrees.

Macrophages are also capable of killing implanted tumour cells without prior immunization. We compared the killing by non-activated or MAF-activated macrophages in an *in vitro* assay. As detailed in **Materials and methods**, [$^{14}$C]-labelled tumour cells were added to bronchoalveolar macrophages, already attached to the bottom of 96-well Falcon microplates, and MAF was added to the culture medium (1:3, v/v). After a 72 h incubation, the supernatants containing the radioactivity released by killed cells were harvested and counted. Figure 1 compares the macrophage activity against R3 tum+ and R9 tum− cells. R9 appeared to be strongly resistant to lysis, even when macrophages were activated. Macrophage activation was confirmed by their increased cytotoxicity against R3 cells. The tum− phenotype of R9 and C8 cells could not be explained by enhanced cytotoxicity of these two immune system effectors.

We have also tried to provoke specific cytotoxicity mediated by T lymphocytes from rats immunized against R9 or C8 cells, either as a direct assay or after a four day stimulation of lymphocytes in the presence of irradiated R9 or C8 tumour cells. The results are not shown since they revealed no cytotoxicity.

In order to demonstrate the role of immunity in the R9 or C8 rejection by immunocompetent Wistar AG rats, we used the Winn assay.

**Figure 1** Macrophage activity against tum$^{+}$ R3 (●, ○) and tum$^{-}$ R9 (■, □). Macrophages were activated (solid symbols) or not (open symbols) by MAF or nocardia extract.

*Adoption of tumour immunity in nude mice*

In genetically athymic nude mice, R9 and C8 cells gave rise to tumour proliferation in one week, when $1 \times 10^6$ cells were injected s.c. When $50 \times 10^6$ T enriched spleen cells from non-immunized rats were mixed with R9 or C8 tumour cells, tumour appearance was delayed 6 days, however all the mice eventually developed tumours (Table V). If T enriched spleen cells were obtained from rats immunized against R9 or C8 tumour cells, tumours did not take in the recipient nude mice, over the 3 month observation time. Moreover, immunization against R9 protected against C8 tumour take, and *vice versa*. These results demonstrate that rats developed immunity against R9 and C8 tumour cells, that T cells were responsible for their rejection and that C8 and R9 shared common antigenic structures.

**Table V** Effect of addition of T enriched spleen cells obtained from normal or immunized rats on the protection of genetically athymic nude mice against R9 or C8 tumour take

| *Origin of T enriched spleen cells* | *Injected cells* | *Tumour take* |
|---|---|---|
| Normal rats | C8 | 4/4 |
| | R9 | 5/5 |
| Rats immunized against R9 | C8 | 0/2 |
| | R9 | 0/5 |
| Rats immunized against C8 | C8 | 0/2 |
| | R9 | 0/5 |

### Discussion

The procedure of drug contact followed by cloning in semi-solid agar designated as the Salmon assay (Salmon *et al.*, 1978) has been used by many experimenters in order to evaluate the sensitivity of stem cells to the cytotoxicity of chemotherapeutic drugs. We have used this procedure to select cell variants resistant to the nitrosourea, CZT. To reinforce cell selection, we repeated this procedure nine times. Similar procedures which consist of repeating drug contacts with the tumour cells were often used with different drugs, leading to the isolation of drug-resistant variants. For example, Giavazzi *et al.* (1983), among others, have obtained adriamycin-resistant cells from a murine fibrosarcoma, by adding progressively increasing doses of adriamycin to the cell culture medium. The originality of our study lies in the fact that we have chosen to select clonogenic and chemo-resistant cells, using a protocol which associated the usual procedure of repeated drug contact with post contact cloning. This procedure was capable of progressively increasing the frequency of clonogenic drug-resistant cells (selective procedure), or increasing the drug resistance of individual clonogenic cells (adaptative mechanism, Laval, 1985). The observation of phenotypic changes in the tumour cells derived from our sarcoma, after treatment by CZT, could explain two previous observations. Firstly, rats grafted with the RMS 9-4/0 tumour, and subsequently treated by CZT developed a greater number of lung metastases than nontreated rats. Secondly, *in vitro* contact of RMS 9-4/0 cells with CZT, increased their cloning efficiency, i.e. induced some tumour cell to express *de novo* a clonogenic efficiency. This *in vitro* procedure for the selection of tumour cell variants, by a selective or adaptive mechanism, could lead to the isolation of highly metastatic and drug resistant variants. However, it excludes the selective pressure exerted by the host.

We report here that the cloning procedure by itself gave rise to tum− variants. Two types of tum− variants were obtained. The R lines after CZT-contact; the C lines without drug contact. These two types of clonogenic cells, colony forming cells (CFCs), were shown to be immunogenic in Wistar AG rats, and tumour cell rejection could be affected by T cells, as was demonstrated by the transfer of immunity to nude mice by T lymphocytes from immunized rats. Moreover, when CZT-resistant CFCs were used to immunize syngeneic recipients, they were able to protect the hosts against metastatic dissemination of the parental cell line, s.c. grafted and growing as a malignant primary tumour. This surprising observation was confirmed by the protective effect of this immunization against pulmonary and liver metastases of tumours induced by the s.c. injection of S4T, a subline isolated from the RMS 9-4/0 parental line. These CZT-resistant clonogenic variants (*in vitro*-selected) had lost their tumorigenicity in the immunocompetent but not in the immunodeficient rat, showing that these cells had acquired immunogenicity. Normal adult rats were immunized with these cells and subsequently grafted with the parental cell line RMS 9-4/0. We observed a reduction in lung metastatic invasion, suggesting that CZT-resistant clonogenic cells share a common antigenicity with cells which are antecedents of the lung metastases of the RMS 9-4/0 tumour and of the liver and lung metastases of the S4T tumour. This new characteristic could be linked to the CZT contact and not to the cloning procedure itself, since these antigenic structures were not present on tumour cells selected only for clonogenicity. However, we cannot exclude the hypothesis that this new antigenicity in CZT-resistant cells has been randomly obtained, by spontaneous mutation of the line more than by CZT-induced mutation. Untreated CFCs (C lines) also lost their tumorigenicity in syngeneic immunocompetent rats, but when used as immunogens, they did not prevent lung metastatic invasion. This phenotypic change is stable in the cell lineage and is probably related to the absence of host immunological selection during the selective pressure. We offer no explanation for this sudden loss of tumorigenicity, or, better still, this sudden acquisition of immunogenicity. This event occurred in one colony (one cell) derived from a clone subpopulation, itself non immunogenic, as if this antigenicity was latent and its repression unstable. On the other hand, the antigenic state seemed to be stable.

The immunogenicity of R9 could be induced by CZT-treatment. One elegant explanation, not yet proven, is that repeated contact with CZT induces the amplification of antigenic structures which are present on tumour cells capable of producing *in vivo* lung or liver metastases. The high malignancy of tumours could be related to a complete loss of antigenicity. However, Van Waes *et al.* (1986) demonstrated the maintenance of one antigenic determinant expressed on progressively more malignant variants derived from UV-induced tumours. Their data led to the conclusion that several structures exist on the poorly malignant, parental line, some inducing immunological rejection mediated by T cell dependent cytolysis, others inducing a T helper mediated mechanism. Their more surprising observation is that the most malignant line (metastatic) which arose spontaneously under immune pressure, induced an immune state in mice, allowing them to reject the graft of the parental tumour but not their own graft. This study clearly showed that this highly malignant tumour also expressed antigenic determinants. The induction of antigenicity on tumour cells by drugs has already been demonstrated. Boon, (1985) has shown that N-methyl-N′-nitro-N-nitrosoguanidine (MNNG) and other mutagens are capable of inducing a high level of mutants in tumour cells and that many of them carry antigenic structures. Some of them are similar to structures present on the parental non-mutated cells, whereas others are specific to each clone. The nature of these structures has not been elucidated, but a tissue-specific structure determined by the tissue origin of the tumour during embryogenesis is one good candidate (Fidler & Nicholson 1976; Frost & Kerbel 1983). Along the same line of thought, Nardelli has indicated that in murine lymphoma cells repeated treatment with drugs *in vitro* generated highly immunogenic sublines (Nardelli *et al.*, 1984). A similar effect was observed after UV irradiation, which was followed by cell changes and the appearance of antigenic structures (Kripke *et al.*, 1978). A hypomethylating drug such as 5-azacytidine was able to provoke similar changes in tumour cells. Moreover, Olsson & Forchhammer (1984) demonstrated that expression of a particular antigen is closely associated with the metastatic potential of tumour cells of the 3 LL adenocarcinoma of C57BL/6 mice. Re-expression of antigens RT1 class I encoded by the major histocompatibility complex (MHC) could also be involved in this newly expressed immunogenicity. The non expression of these structures at the surface of the tumour cells (reviewed by Goodenow *et al.*, 1985) or the abnormality of the balance between the expression of the two major components, as related by Katzav *et al.* (1985) could be closely related to their tumorigenicities and their metastatic abilities. Experiments are in progress to elucidate if these cells diversely express these RT1 class I antigens.

To the effectors of the immune system such as NK lymphocytes and activated macrophages selected cells appeared to be close to normal cells (self). Both tum− cell lines became progressively resistant to NK− and macrophage-induced cytotoxicity. The loss of tumorigenicity cannot be explained by this fact. Finally, using these procedures we have revealed two types of antigenicity. A common antigen was present on R9 and C8 tum− cells, which was responsible for a cross-linked protection demonstrated in nude mice by the Winn assay. Though we have shown that T cells transferred the immunological protection, we have not succeeded with *in vitro* T-cell mediated cytotoxicity (negative results, not shown). A different antigen was present only on R9 cells, and not on C8 cells. This latter antigenicity was present on metastatic cells and CZT-treated cloned cells. Moreover, these immunizing structures were also on CZT-resistant cells selected *in vivo*. Concerning the fundamental aspects of the biology of metastases, this observation could be important in establishing a link between resistant parental cells and metastatic cells. In our studies, these two subpopulations had common properties which led to their selection. Metastatic dissemination of tumours involves cellular subpopulations which could be more resistant to the chemotherapeutic drugs than the cell populations of the primary lesion. An alternative could be that the metastatic cells could be more susceptible to the mutagenic effect of drugs, rendering these cells subsequently more resistant. The evidence of a common antigenicity leads us to believe that the same cell can have the resistant and metastatic phenotypes and that cloning in semi-solid medium could isolate this cell.

We would like to thank V. Lascaux and Y. Rolland for their excellent technical help, Mrs P. Blanchin for secretarial assistance and J. Jacobson for the improvement of the manuscript.

### References

BOON, T.& KELLERMAN, O. (1977). Rejection by syngeneic mice of cell variants obtained by mutagenesis of a malignant teratocarcinoma cell line. *Proc. Natl. Acad. Sci.* (*USA*) **74,** 272.

BOON, T. (1985). Tum– variants: immunogenic variants obtained by mutagen treatment of tumor cells. *Immunol. Today*, **6,** 307.

CHEN, T.R. (1977). *In situ* detection of mycoplasma contamination in cell culture by fluorescent Hoechst 33258 stain. *Exp. Cell Res.*, **104,** 255.

EVANS, R. (1986). The immunological network at the site of tumor rejection. *Biochem. Biophys. Acta.*, **865,** 1.

FIDLER, I.J. & NICOLSON, G.L. (1976). Organ selectivity for implantation survival and growth of B16 melanoma variant lines. *J. Natl. Cancer Inst.*, **57,** 1199.

FIORETTI, M.C., BIANCHI, R., ROMANI, L. & BONMASSAR, E. (1983). Drug-induced immunogenic changes of murine leukemia cells: dissociation of onset of resistance and emergence of novel immunogenicity. *J. Natl. Cancer Inst.*, **71,** 1247.

FROST, P. & KERBEL, R.S. (1983). On the possible epigenetic mechanism(s) of tumor cell heterogeneity. *Cancer Metastasis Rev.*, **2,** 375.

GIAVAZZI, R., SCHOLAR, E. & HART, I. (1983). Isolation and preliminary characterization of an adriamycin-resistant murine fibrosarcoma cell line. *Cancer Res.*, **43,** 2216.

GOODENOW, R.S., VOGEL, J.M. & LINSK, R.L. (1985). Histocompatibility antigens on murine tumors. *Science*, **230,** 777.

KATZAV, S., SEGAL, S. & FELDMAN, M. (1985). Metastatic capacity of cloned T 10 sarcoma cells that differ in H-2 expression: inverse relationship to their immunogenic potency. *J. Natl. Cancer Inst.*, **75,** 307.

KRIPKE, M.L., GRUYS, E. & FIDLER, I.J. (1978). Metastatic heterogeneity of cells from an ultraviolet light-induced murine fibrosarcoma of recent origin. *Cancer Res.*, **38,** 1962.

LAVAL, F. (1985). Repair of methylated bases in mammalian cells during adaptive response to alkylating agents. *Biochimie.*, **67,** 361.

NARDELLI, B., CONTESSA, A.R., ROMANI, L., SAVA, G., NISI, C. & FIORETTI, M.C. (1984). Immunogenic changes of murine lymphoma cells following *in vivo* treatment with aryl-triazene derivatives. *Cancer Immunol. Immunother.*, **16,** 157.

OLSSON, L. & FORCHHAMMER, J. (1984). Induction of the metastatic phenotype in a mouse tumor model by 5-azacytidine and characterization of an antigen associated with metastatic activity. *Proc. Natl. Acad. Sci.* (*USA*), **81,** 3389.

PAUWELS, C., REBISCHUNG, J.L., JASMIN, C. & POUPON, M.F. (1985). Enhanced cloning efficiency of murine rhabdomyosarcoma cells after chlorozotocin treatment: relationship with enhanced lung metastasis. *J. Natl Cancer Inst.*, **74,** 817.

POT-DEPRUN, J., POUPON, M.F., SWEENEY, J.L. & CHOUROULINSKY, I. (1983). Growth, metastasis, immunogenicity and chromosomal content of a nickel-induced rhabdomyosarcoma and subsequent cloned cell lines in rats. *J. Natl. Cancer Inst.*, **71,** 1241.

POUPON, M.F., PAUWELS, C., JASMIN, C., ANTOINE, E., LASCAUX, V. & ROSA, B. (1984). Amplified pulmonary metastases of a rat rhabdomyosarcoma in response to nitrosourea treatment. *Cancer Treat. Rep.*, **68,** 749.

PRICE, J.E., SYMS, A.J., WALLACE, J.S., FLEMING, K.A. & TARIN, D. (1986). Cellular immortality, clonogenicity, tumorigenicity and the metastatic phenotype. *Eur. J. Cancer Clin. Oncol.*, **22,** 349.

SALMON, S.E., HAMBURGER, A.W., SOEHNLEN, B. & 4 others (1978). Quantification of differential sensitivity of human-tumor stem cells to anticancer drugs. *N. Engl. J. Med.*, **298,** 1321.

SWEENEY, F.L., POT-DEPRUN, J., POUPON, M.F. & CHOUROULINKOV, I. (1982). Heterogeneity of the growth and metastatic behavior of cloned cell lines derived from a primary rhabdomyosarcoma. *Cancer Res.*, **42,** 3776.

VAN PEL, A., VESSIERE, F. & BOON, T. (1983). Protection against two spontaneous mouse leukemias conferred by immunogenic variants obtained by mutagenesis. *J. Exp. Med.*, **157,** 1992.

VAN WAES, C., URBAN, J.L., ROTHSTEIN, J.L., WARD, P.L. & SCHREIBER, H. (1986). Highly malignant tumor variants retain tumor-specific antigens recognized by T-helper cells. *J. Exp. Med.*, **164,** 1547.

WINN, H.J. (1961). Immune mechanisms in homotransplantation. II. Quantitative assay of immunologic activity of lymphoid cells stimulated by tumor homografts. *J. Immunol.*, **86,** 228.